# Spatial Simulations in Systems Biology: From Molecules to Cells

**DOI:** 10.3390/ijms13067798

**Published:** 2012-06-21

**Authors:** Michael Klann, Heinz Koeppl

**Affiliations:** Automatic Control Laboratory, ETH Zurich, Physikstrasse 3, 8092 Zurich, Switzerland

**Keywords:** Brownian dynamics, agent-based modeling, diffusion-controlled reactions, fractal kinetics, nonlinear diffusion, spatial-temporal dynamics

## Abstract

Cells are highly organized objects containing millions of molecules. Each biomolecule has a specific shape in order to interact with others in the complex machinery. Spatial dynamics emerge in this system on length and time scales which can not yet be modeled with full atomic detail. This review gives an overview of methods which can be used to simulate the complete cell at least with molecular detail, especially Brownian dynamics simulations. Such simulations require correct implementation of the diffusion-controlled reaction scheme occurring on this level. Implementations and applications of spatial simulations are presented, and finally it is discussed how the atomic level can be included for instance in multi-scale simulation methods.

## 1. Introduction

At the beginning of this century Tomita [1] wrote the article “Whole-cell simulation: a grand challenge of the 21st century”. With the present contribution, we want to review how far we progressed after one decade. Clearly, the cell is not an unstructured bag of enzymes but highly organized in space [2]. Some structures or compartments can be readily seen under the microscope [3], others emanate more subtle, for instance by the clustering of molecules leading to a microcompartmentalization of the cell [4–6]. This organization gives rise to spatial-temporal dynamics in the cell [7–9].

Depending on the chosen description of the cell, the cellular state is encoded by the amount of molecules of each molecular species, but also by the distribution of molecules in the cell and the actual state (e.g., phosphorylation level) or conformation of each molecule [3]. Thus also on the molecular level the spatial organization of the atoms or the electron density respectively controls the state of the cell [13]. If we want to track how a conformational change of a single molecule can influence the state of the cell, we therefore have to bridge scales from 10^−10^ to 10^−5^ m and 10^−12^ to 10^3^ s [14].

For each individual level many simulation methods exist, as reviewed in [15–19]. For instance, the overall temporal dynamics in the cell can be simulated with ordinary differential equations (ODE), and the spatial component can be included if partial differential equations (PDE) are used [7]. In order to include stochasticity due to low particle numbers, stochastic differential equations (SDE) have to be used, and Gillespie [20] developed an efficient way to simulate the outcome of the chemical master equation, describing the evolution of the population of molecule species. On the molecular level, the dynamics can be investigated in detail using molecular dynamics simulations (MDS) [21,22].

Despite the increasing computational power of workstations and supercomputers, simulation of the whole cell at the atomic level remains prohibitively expensive. For example a small yeast cell contains “only” 50 million proteins [23], however there are more relevant molecules (DNA, RNA, lipids, metabolites), and especially all the ions and water molecules in the cell. In addition, each macromolecule itself consists of thousands of atoms, such that the number of states for each object in the simulation has to be reduced for a simulation [24]. The cell could for instance be simulated at a more coarsely grained level, while only relevant parts could still be tracked on the atomic level in a multi-scale simulation [25].

Particle-based methods can be a useful tool to analyze cellular dynamics. The particles in these methods can represent atoms as in MDS, molecular subunits [26,27], whole molecules [28], sub-volumes of the cell [29,30] or even a whole cell each [31,32]. The particle interactions and actions then have to be defined according to the chosen level of granularity. Especially if the actions of the objects in the simulation are determined by their own internal state or dynamics, e.g., if they represent individual cells, the term cellular-automata or agent-based becomes more appropriate [33].

**Figure 1:**
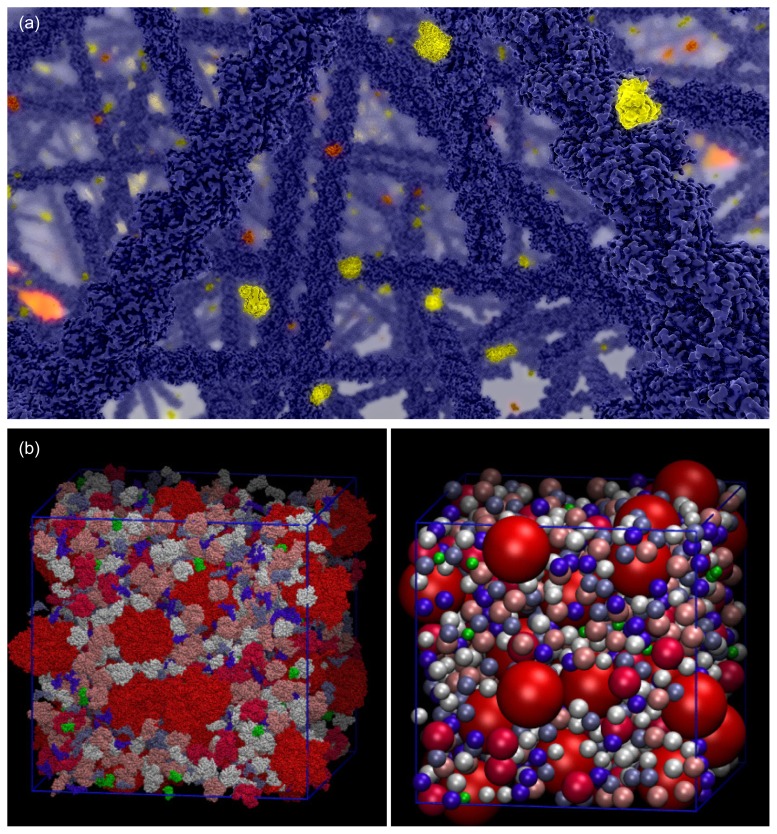
(**a**) Visualization of the cytoplasm from a Brownian dynamics simulation including cytoskeleton filaments and just the signaling molecules of one pathway. For visualization, all molecules and cytoskeleton filaments have been replaced by their atomic structure and rendered by raytracing (ScienceVisuals [10,11]); (**b**) Physiological level of crowding, *i.e*., a representative molecular size distribution and abundance, modeled either with spheres or real molecule shapes by Ando and Skolnick [12] in a cubic subvolume of the cytoplasm. Reproduced with permission of PNAS. Due to the high density of molecules it is impossible to see through the cytoplasm. These crowding conditions affect diffusion and reactions in the cell.

In the present review we first describe a particle-based mesoscopic level which can serve as a basis for whole cell simulations (cf. Figure 1). Then we show possible applications of such simulations and finally we discuss how it can be extended in several directions in a multi-scale approach.

## 2. The Mesoscale Level

In order to track the dynamics for instance of signaling molecules in the cell, each molecule is represented by a particle in the simulation. This level is often called mesoscale, because it is between the microscopic atomic resolution of MDS and the macroscopic resolution of ODE/SDE/PDE methods. The following sections describe how the molecules move and interact in the cell at this level.

### 2.1. Diffusion in the Cell

Obviously the solvent cannot be included explicitly in a whole cell simulation [34]. Langevin dynamics describe the motion of particle *j* (position vector ***x****_j_*) with mass *M_j_* in such a case based on [22]

(1) $$M_{j}\frac{{\text{d}}^{2}x_{j}}{\text{d}t^{2}}=-\nabla U(x_{j})-\gamma_{j}M_{j}\frac{\text{d}x_{j}}{\text{d}t}+\sqrt{2k_{B}T\gamma_{j}M_{j}}R(t)$$

within the interaction potential *U*(***x***), at temperature *T* and subject to friction *γ_j_* with the Boltzmann constant *k_B_* and (zero mean) white noise vector ***R***(*t*), which represents the force induced by collisions with solvent molecules. According to the fluctuation-dissipation theorem the energy added by ***R*** is dissipated by *γ_j_* such that the system reaches and fluctuates around *T* [22].

The overdamped case (*i.e*., the damping is so strong that inertia can be neglected) reduces Langevin dynamics to Brownian dynamics

(2) $$\frac{\text{d}x_{j}}{\text{d}t}=-\nabla U(x_{j})/\zeta_{j}+\sqrt{2D_{j}}R(t)$$

where the damping is expressed by *ζ_j_* = *γ_j_M_j_* and the diffusion coefficient *D_j_* = *k_B_T/ζ_j_*. Especially if the particles are connected, e.g., because they represent sub-segments of a cellular filament, great care has to be taken on the definition of the corresponding interaction potential *U* between them [26,27,31,35].

The authors understand the importance of all intramolecular forces, and especially electrostatic and hydrodynamic interactions [12,36–38]. Still, if all forces are neglected, the Brownian dynamics motion can be easily integrated with a discrete time EulerMaruyama scheme as a random walk [39]:

(3) $$x_{j}(t+\Delta t)=x_{j}(t)+\sqrt{2D_{j}\Delta t}\xi$$

with ***ξ*** a three-dimensional zero mean Gaussian random variable with unit variance (the difference of the Wiener process *R*(*t* + Δ*t*) − *R*(*t*) ~ 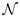
(0*,*Δ*t*); for convenience the Δ*t* has been included in the square root in Equation (3) such that readily available standard normal random numbers can be used).

As long as intramolecular potentials/forces are included, they prevent the overlap of particles [40]. Otherwise the new position will have to be rejected if the particle jumped to an excluded position [41–43]. Thus intracellular structures and all the other molecules hamper the diffusion of each molecule. While the expected mean squared displacement (MSD)

(4) $$\langle {\left(x(t)-x(0)\right)}^{2}\rangle =2dDt$$

should depend linearly on *D*, *t* and the dimension *d*, it is found that it only grows with a reduced *D*′ if diffusion is hindered by other objects. Several Monte Carlo or Brownian dynamics simulations evaluated the effect of fixed crowding structures on the diffusion of tracer molecules in the cytoplasm or cellular compartments [30,42,44–48]. Especially the intracellular matrix built by the cytoskeleton and proteins bound to it (microtrabecular lattice) looks like porous media and can cover up to 20% of the intracellular volume [49,50]. Diffusion through such structures leads to similar effective diffusion coefficients [42,43,51], like the predicted *D/D*′ from Weissberg [52] for porous media (Note that the comparison can require volume averaging [53]). In general, bigger molecules are hindered more strongly in their mobility, and at the same excluded volume fraction many small obstacles lead to a stronger hindrance compared to a few big ones [37,42,49]. Large diffusing objects with respect to the mesh size of the cytoskeleton network can be caged/trapped in these meshes and are restricted to their subvolume, which means that their MSD is limited [42,49]. Several theories have been developed to describe diffusion through polymer solutions like the cytoplasm [54–58]. The complexity of the cytoplasm requires to use approximations or to rely on empirical formulas to compute the expected effective diffusion depending on the molecular radius (and the shape), the free/excluded volume fraction, and the size (distribution, shape...) of the crowding objects [49,59,60].

Other studies also revealed subdiffusion or at least transient anomalous behavior [58]. This means, that the MSD did grow only *α t^α^* with *α <* 1 [40,42,61], a fact that can also be observed with Fluorescence Correlation Spectroscopy (FCS) [40] The degree of subdiffusion also is a measure of the crowding level in the cell [40,62]. Such studies with mobile crowding objects require much more computation time than simulations with static objects [63]. In addition, the obstacles hinder the test molecules, while the test molecules influence the mobility of the obstacles. This feedback requires to model molecular crowding as exactly as possible in order to analyze the effective diffusion in the cell, for instance by using an obstacle size and abundance distribution similar to the *in vivo* conditions [41,60]. Table 1 shows relations between molecular weight and hydrodynamic radius of the molecules as used in such detailed simulations. Thus mesoscale simulations can be used to calculate the mobility of biomolecules on the cellular level, although techniques like MDS [59], dissipative particle dynamics [64] or multiparticle collision dynamics [65] have been employed as well. Connected Brownian particles can also represent polymer chains like in the *Brownmove* package [34]. Such simulations allow the analysis of filament shapes and diffusion [26,66] and the rheology of biopolymer networks [27,29,35,67]. Simulations with motor proteins furthermore show how network patterns emerge [68].

In addition to undirected diffusion, cells also contain motor proteins that can pull molecules along the cytoskeleton [73]. This effect can either be included in the model as a general drift term in *U*(***x****_j_*) or by switching the transport mode if molecules bind to a cytoskeleton filament and moving the molecules linearly in their direction [74,75]. Of course also the action and properties of the motors have been studied extensively in detail using MDS [76,77]. Eventually, also the effect of the dynamic cytoskeleton and cell shape has to be included in the simulations [78].

Most biomolecules are not inert, which means that they interact with the others. This interaction is for instance required to bind to the motor proteins for the directed transport. But molecules can also unspecifically bind to the cytoskeleton [79]. This sequesters molecules from the cytosol making it less crowded, forms the microtrabecular lattice in the cell and can co-localize molecules which belong together [4]. Transient binding of course reduces the mobility of the molecules, because bound molecules are immobile [79,80].

### 2.2. Reactions in the Cell

Reactions between molecules require that these molecules come into contact. Since the motion of the molecules is mostly determined by diffusion, all reactions between molecules are diffusion controlled. Smoluchowski [81] calculated the collision “rate constant” of spherical molecules (here between molecule *i* and *j* with the respective radii *r_i_, r_j_* and diffusion coefficients *D_i_, D_j_* ):

(5) $$\gamma_{ij}=4\pi (r_{i}+r_{j})(D_{i}+D_{j})$$

and obviously no reaction can occur with a faster rate than this collision process. For example two molecules with *r_i_* = *r_j_* = 2.5 nm (corresponding to a molecular weight of about 25 kDa, cf. Table 1) and diffusion coefficient *D_i_* = *D_j_* = 80 *μm*^2^/s have a diffusion limit for their reaction of *γ_ij_* = 6.05 *×* 10^9^ M^−1^s^−1^. Probably enzymes cannot react that efficiently, and thus this limit is in agreement with observed biomolecular reaction rate constants.

Let *k_ij_* denote the macroscopic (observable) reaction rate constant between molecule *i* and *j*, *i.e*., the rate constant which is also used in ODE/PDE models, assuming (locally) well mixed conditions. Obviously, the microscopic situation of two molecules being in contact is fundamentally different. The corresponding microscopic rate constant *κ_ij_* describes the fraction of collisions that lead to reactions. These microscopic rates can be observed e.g., in MDS or BD where the reaction dynamics is solely described by interaction potentials [82–84]. In three dimensions, the macroscopic rate constant related to the microscopic one is given by [85,86]

(6) $$\frac{1}{k_{ij}}=\frac{1}{\gamma_{ij}}+\frac{1}{\kappa_{ij}}$$

Therefore, if diffusion is fast enough (1*/γ_ij_* → 0), the microscopic rate equals the macroscopic one. The possible difference between these rates should however be considered, when comparing MDS and ODE models: while ODE models use the macroscopic descriptions, in MDS the simulation volume is so small that the molecules are basically always in contact, *i.e*., the microscopic description is more applicable [82].

The physiological conditions in the cell can have a strong effect on such reactions, and render them different from *in vitro* kinetics obtained in test tubes [87,88]. Of course all kinds of co-factors and allosteric modifiers can alter the functionality of an enzyme and the “pressure” by the surrounding crowding molecules can change the molecular conformation [87,89]. But in addition molecular crowding alters the collision frequency of the molecules [88].

Let us assume we have *N_i_* and *N_j_* molecules in the cell with volume *V* and the reaction is described by mass action kinetics. Then the (ODE) reaction rate is given based on the concentrations *c_m_* = *N_m_/V*, where *m* is species *i* or *j: r_ij_* = *k_ij_c_i_c_j_* (the stochastic propensity is *a_ij_* = *k_ij_N_i_N_j_/V* respectively). This rate/propensity changes due to excluded volume effects, because only a smaller volume is accessible for the molecules. The molecules effectively have a higher concentration, but based on the original volume, the rate constant will appear to be increased (compared to the *in vitro* case in diluted conditions) [80,88].

In contrast, the reduced diffusion leads to a reduced collision frequency, as can be seen from Equation (5). As long as we assume that the microscopic reaction rate constant remains constant (no allosteric change due to crowding), the macroscopic reaction rate constant appears to be decreased (cf. Equation (6)) [80]. Moreover, if diffusion becomes nonlinear due to crowding, the reaction rate constant can appear to be time dependent. Several *in silico* studies have observed such fractal kinetics for instance due to compartmentalization or crowding, when the obstacles hamper the restoration of the well mixed molecule distribution that is critical for spatially non-resolved population level descriptions of the dynamics [17,90,91]. Several studies therefore targeted reaction diffusion processes under crowding [37,41,63,80,92]. Further studies also investigated reactions within tubular interconnected reaction compartments such as the ER [93,94] or reactions that are enhanced by transport with motor proteins [95,96].

Finally it should be noted that the dimensionality of the system plays an important role for reactions. The collision rate constant *γ_ij_* has different expressions in one, two, and three dimensions [97,98]. Thus it was for instance discussed whether signaling molecules are bound to the plasma membrane to make use of the resulting 2D kinetics [5,99]. Also the membranes can be crowded and structured, and in 2D caging effects occur much faster, leading to complex reaction diffusion behavior [100].

### 2.3. Reactions in the Simulation: Implementation Issues

Especially for the description of biological processes several nonlinear reaction schemes and kinetics have been developed. Examples are Michaelis–Menten and Briggs–Haldane enzyme kinetics [101] but also Hill kinetics to describe cooperativity and additional effects [102]. These schemes provide one reaction rate for the whole process (under the quasi-steady state assumptions [103]), while the individual steps of the process are described by mass action kinetics [101]. The effective rate constant for Michaelis–Menten kinetics describing the process

$$E+S\overset{k_{f}}{\underset{k_{r}}{⇄}}ES\overset{k_{cat}}{\to }E+P$$

or in terms of balance Equations

$$-\frac{\text{d}[S]}{\text{d}t}=\frac{\text{d}[P]}{\text{d}t}=k([S])[E][S]$$

(with [*E*] = *const*) depends on the overall current substrate concentration [*S*] [101]

(7) $$k([S])=\frac{k_{cat}}{K_{m}+[S]}$$

and the two parameters *k_cat_* and *K_m_*. This means that information is lost from the original description with three reactions (*K_m_* = (*k_r_* + *k_cat_*)*/k_f_* ). With respect to particle based simulations, where reactions are triggered by collisions of molecules, this means that each colliding pair would have to know the current overall concentration [*S*] in order to determine the rate applicable to its own reaction, which is unphysical. Therefore the authors suggest to only use plain mass action kinetics in particle based simulations on the molecular level.

The corresponding reaction rates and rate constants for mass action kinetics count/determine the (average) number of events per time unit. These rates can also be observed in MDS or BD where the dynamics is solely described by interaction potentials [82–84]. However with respect to a whole cell simulation, an event based algorithm using the macroscopic rate constants as parameters is more desirable.

The highest order of reaction which can be reasonably modeled are second order/bimolecular reactions, because third order reactions mean that three particles collide simultaneously. This is extremely unlikely and mostly such reactions form intermediary dimers which collide with the third particle instead. Accordingly third order reactions should be modeled as a set of second order reactions.

First order reactions with the rate *r_i_* = *k_i_c_i_* or propensity *a_i_* = *k_i_N_i_* require to execute (on average) *a_i_* events per time unit on the population level. In a discrete time particle based simulation, each particle will react in the time interval (*t, t*+Δ*t*] with probability [28]

(8) $$P_{i}=1-\text{exp }(k_{i}\Delta t)\approx k_{i}\Delta t$$

In the simulation therefore in each time step and for each particle involved in this reaction a uniform random number *ξ*_1_ has to be compared with *P_i_*. The particle will react if *ξ*_1_ ≤ *P_i_* with *ξ*_1_ *~* 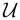
[0*,* 1] (and of course *P_i_* *<* 1).

A lot of random numbers and computation time can be saved, if instead the time to the next event is pre-computed when the molecule is created in analogy to Gillespie’s stochastic simulation algorithm [20]. The waiting times *t_i_* for all molecules are exponentially distributed (Exp(*k_i_*)), and in this context it is worth noting that the minimum time min (*t_i_*) *~* Exp(*k_i_N_i_*). Thus this description is compatible with the chemical master equation description on the population level. The trade-off is that all individual waiting times have to be stored and ordered, executed in their sequence, and especially updated if other processes interfere with the assigned reaction channel [104–106].

Zero order reactions ∅ → *I* with rate *r_i_* = *k_i_*^(0)^ or propensity *a_i_* = *k_i_* can be implemented similarly. New molecules of the product species *I* are created (out of nothing) after the waiting time min (*t_i_*) *~* Exp(*k_i_*), where the position of the new molecule is drawn from a given spatial distribution. Alternatively they can be generated by a first order reaction based on a dummy species *D* with fixed concentration *c_D_* and the scheme *D* → *D* + *I* and rate constant *k*′ = *k_i_*^(0)^*^/^c_D_* [75]. Since the number of molecules is changing, a buffer of empty particles is required for the simulation [107].

**Figure 2:**
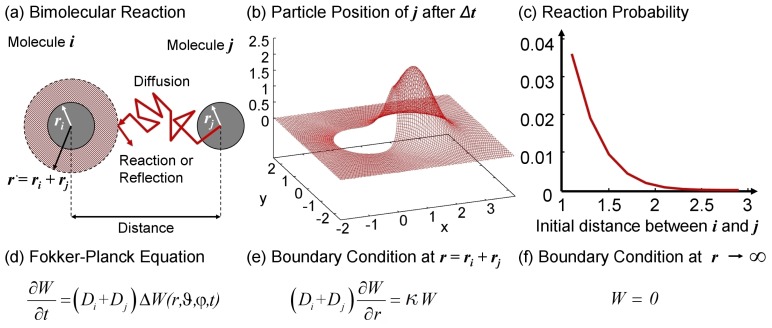
(**a**) Two diffusing molecules can collide and will be reflected if they do not react; (**b**) Corresponding probability density function (pdf) for the distance of *j* relative to *i* as described by the Fokker–Planck equation; (**c**) Reaction probability depending on the initial distance; (**d**)–(**f**) Fokker–Planck equation and boundary conditions. The pdf for the distance *r* between two diffusing molecules as described by (**d**) starting from *W*(0) = *δ*(***x****_i_* − ***x****_j_*) is shown in (**b**). In this description, particles react if they diffuse “into” the reaction partner, which is accounted for by the flux across the collision surface and depends on the microscopic rate constant *κ*. Therefore the boundary condition (**e**) is partially reflecting. For *κ* = 0 (**e**) becomes completely reflective, describing two inert particles. Note the “blister” in (**b**) which deforms the normal distribution at the boundary caused by the reflection. Due to incomplete reflection, the total probability ∫ *WdV <* 1. The loss corresponds to the reaction probability. Hence the reaction probability (**c**) for a given initial distance is found as $P_{FP}^{reaction}=1-\int_{r^{*}}^{\infty }WdV$ [75,109,114].

Second order reactions require a more elaborate description [39]. As stated above, bimolecular reactions are triggered by (diffusion-controlled) collisions of molecules, *i.e*., the simulation has to identify the pairs currently in contact. In a discrete time simulation the number of pairs which will be found in a time interval thus depends on Δ*t*, *i.e*., how frequently it is checked. The true number of collisions remains unknown, because the path of the molecules between *t* and *t*+Δ*t* is undetermined. Clifford and Green [108] suggested a Brownian bridge to interpolate the paths of the molecules in order to find all collisions. Lapin *et al*. [109] in turn used the Fokker–Planck equation in order to determine the probability of a reaction within Δ*t* given the current distance *||****x****_i_*(*t*) − ***x****_j_*(*t*)*||*, microscopic rate constant *κ_ij_* and diffusion coefficient *D_ij_* = *D_i_* + *D_j_* (see Figure 2). Note that in this framework the new particle position is not updated according to Equation (3) but according to the probability density distribution (pdf) such that the particles will never overlap. Obviously this description has to be applied only if the particles are within the interaction range. For separated particles, the pdf converges to the normal distribution.

Again, most steps will not result in reactions. Similarly to the treatment of first order reactions using Gillespie’s approach also here the time to the event can be computed instead of checking and wasting random numbers each step. The reaction-diffusion equation for the probability density function (see Figure 2) can be solved analytically using Green’s functions from which the probability of a reaction between particle *i* at ***x****_i_*(*t*) and *j* at ***x****_j_*(*t*) and if so also the time *t* + τ^*^ and position x^*^ of it can be deduced [110]. The resulting Green’s function reaction dynamics (GFRD) is in agreement with the corresponding analytical solutions for diffusion limited reactions [111–113].

The challenge in the underlying theory is that it can only be solved analytically for pairs, but in the cell we have much more molecules [115]. Therefore the step length or event horizon respectively in GFRD has to be set such that no additional molecule enters the vicinity of a pair [110]. For low particle concentrations, in turn, GFRD allows a great speedup of the simulation.

Several methods aim at a simpler model based on a critical radius r_ij_^*^ (*k_ij_,* Δ*t*) such that molecules will react if their distance is smaller than r^*^_ij_ [28,80,116–118]. Such an algorithm will compute reactions faster than GFRD or the Fokker–Planck method because it does not need complex look-up tables. However, in order to reach the same accuracy, a shorter time step than in GFRD might have to be chosen, which requires more steps in total to complete the simulation [111]. In addition, the computation of a suitable critical radius r^*^_ij_(*k_ij_,*Δ*t, . . .* ) is not straightforward, requiring numerical calculations before the simulation can start and can be implementation dependent [28,39,118].

A stable and rather simple derivation is given in [80] for particles that can overlap:

- set the critical reaction radius to the physical collision radius
  $$r_{ij}^{*}=r_{i}+r_{j}$$
- and execute reactions for particles with *||****x****_i_*(*t*) − ***x****_j_*(*t*)|| ≤ r^*^_ij_ with probability
  (9) $$P_{ij}^{*}=\frac{\kappa_{ij}\Delta t}{4\pi {\left(r_{i}+r_{j}\right)}^{3}/3}$$

Obviously P^*^ *_ij_* *<* 1, which limits Δ*t*. From tests we found that this approach works reliably up to P^*^_ij_ *<* 0.2 even for significant degrees of diffusion control. Theoretical limits of this approach or respective correction factors for the reaction probability are calculated in [39].

It is especially important for this approach to work correctly, that the critical radius is the same as the radius used to determine the diffusion limit *γ_ij_* Equation (5) in order to obtain the expected macroscopic rate constant. Note, that bimolecular rate constants (for reactions in 3D volumes) have units *volume/time* (conversion from *M*^−1^*s*^−1^ to *μm*^3^*/s* using 10^15^*/*6.022 *×* 10^23^ M*μm*^3^). This can be interpreted as reaction volume per molecule and time [116]. The present approach uses the ratio of the microscopic reaction volume to the interaction volume as reaction probability given that a collision has happened, which is a mechanistic analogy to diffusion controlled reaction scheme where the microscopic rate constant describes how efficiently collisions are turned into reactions. Based on this formalism also reactions with other geometries can be defined, for instance binding/adsorption to membranes or cytoskeleton filaments [75]. If the reacting objects are not allowed to overlap, obviously the reaction volume has to be wrapped around the collision radius rather than being distributed within their collision radius. If the thickness of this reaction volume layer compared to the collision radius is negligible, the constraint that the critical reaction radius should match the collision radius in Equation (5) will be satisfied without using a reaction probability [75].

Reversible reactions like *A* + *B* ⇌ *C* require a special treatment in this context [28,111,119,120]. Not only the association but also dissociation can be diffusion limited [121]. For strong association and slow diffusion, a pair of just dissociated particles can hardly diffuse away from each other before they will recombine. In order to obey detailed balance therefore also a microscopic dissociation reaction has to be introduced such that the dissociation constant

(10) $$K_{d}=\frac{\left[A][B\right]}{\left[C\right]}=\frac{k_{c}}{k_{ab}}=\frac{\kappa_{c}}{\kappa_{ab}}$$

is preserved [111]. Similar to the microscopic binding reaction rate constant, which was introduced above, the microscopic rate constant *κ_c_* counts all dissociation events, while the macroscopic rate constant *k_c_* only includes the successful events where the molecules could escape from each other and reach a macroscopically observable distance. From Equation (10) follows that the backward first order reaction rate constant has the same scaling like the forward second order rate constant, which is determined by Equation (6) in 3D: *κ_c_* = *k_c_* *× γ_ab_/*(*γ_ab_* *− k_ab_*).

In order to reach the correct geminate recombination rate also the initial condition of the new pair *A* and *B* upon dissociation has to be chosen carefully. In the *Smoldyn* framework of Andrews *et al*. [28,119,120] as well as in GFRD [110,111] therefore an unbinding radius outside of the critical binding/reaction radius has to be used. Since space and time are related, the onset of the geminate recombination process can likewise be delayed by blocking reactions for particles that just reacted. If the particles *A* and *B* are placed at identical positions ***x****_A_* = ***x****_B_* (= ***x****_C_* from which they were created from) this delay term is

(11) $$\tau_{i}=\frac{1}{10}{\left(r_{a}+r_{b}\right)}^{2}/(D_{a}+D_{b}),\;\;\;i=a,b$$

which is the maximum likelihood of the (diffusion-controlled) escape process [122]. Placing the molecules overlapping can be necessary in simulations with crowding objects, because the dissociation *C* → *A* + *B* might occur at a position where no valid separated positions for *A* and *B* exists. Note that such crowding structures which reduce diffusion keep the molecules together and therefore push equilibrium towards the associated *C* state.

Depending on the implementation for parallelized execution in multi-threaded CPU or GPU applications anyway a flag might be necessary to execute each reaction for each molecule not more than once [107]. This flag can be implemented using the same memory like the necessary functional delay term Equation (11) by preventing that molecules react in any reaction, which have a delay *> t* assigned, and setting the minimum delay for each particle that reacted to *t* + 0.5Δ*t*. Note that the global delay for reversible reactions does not affect the overall reaction process because it is so small [122].

Such a delay term can also be used in order to mimic Michaelis–Menten enzyme kinetics[75,107]. In the reduced reaction scheme *E* +*S* → *E* +*P* the enzyme *E* is blocked for a certain time *τ* after each reaction, leading to a saturation of the reaction rate. Let us use the standard notation *k_cat_* and *k_r_* for the first order reactions and *k_ES_* instead of *k_f_* for the second order *E* +*S* → *ES* reaction [101]. If only *k_cat_* and *K_m_* are given, *k_ES_* can be set to *k_ES_* = *k_cat_/K_m_* [75,107].

The delay term introduced here for reversible reactions is also in agreement with the overdamped Langevin regime discussed here. Upon dissociation we would expect that the two molecules have to pick up and maintain impulses which separate them for a given time. The reversion of these impulses such that the molecules can re-associate certainly requires additional time, which is not covered by the plain random walk implementation. In the detailed dissociation process, the molecule will first have to accumulate the necessary energy in order to overcome the potential energy of the bond and to dissociate. Once it has this energy, the fate of the molecule is already dissociation, but only with a delay the bond will be actually broken. A similar discussion, however in terms of the adjoint dissociation distance, is found in Andrews *et al*. [120].

Future work has to find ways to include more detailed description on such a simplified simulation scheme or at least bridge the detailed and simplified approach. Especially nonspherical molecules and specific binding sites on the surface of the molecules are relevant for biomolecular reaction kinetics [123–128].

### 2.4. Performance and Accuracy

The performance of a particle based simulation strongly depends on the efficiency of the used pair finding and collision detection algorithms [129]. In order to cover a sufficient time span with the simulation, also a large Δ*t* is desirable to reduce the number of steps. Constraints on the accuracy limit the choice of Δ*t*.

Within a crowded intracellular environment the random walk steps have to comply with the boundaries of the obstacles. Steps can either be rejected (completely or retried) if they end in an obstacle [42,43,75,80] or reflected [110,130]. However, if complex surfaces are considered, calculation of the reflected endpoint can become time consuming and suffer from numerical imprecisions. Especially for densely crowded regions, one step might interfere with more than one obstacle. Thus obstacle density and size give upper bounds for the step length, similar to the condition in GFRD that a particle must interact with at maximum one particle within the next time step [110]. As such, the step size could be adjusted individually for each particle depending on its distance to the closest object.

The random number distribution for diffusion simulations (Equation (3)) should be a normal distribution with *μ* = 0, *σ*^2^ = 1. These random numbers can be obtained by several algorithms based on uniformly distributed pseudo random numbers [131]. From our observations, a normally distributed random number costs at least 20% more than a uniformly distributed one. However, the repeated application of the uniform distribution will lead to a normal distribution due to the central limit theorem [132], and the convergence sufficient for simulation occurs within 5 steps. The authors also are very faithful that a repeated rejection sampling of steps from the uniform distribution will converge to a reasonable distribution within a crowded intracellular environment, even without using complex reflection calculations [130].

In addition, the uniform distribution does not have long tails. For a *σ*^2^ = 1 uniform random variable the distribution has to have a width of $\sqrt{12}$. Taking into account the scaling factor of Equation (3), the maximum step length $\Delta x_{max,u}=1/2\sqrt{12\times 2D\Delta t_{u}}=\sqrt{6D\Delta t_{u}}$. The maximum step length in space becomes important for instance if only the end point of a step is checked with all obstacles/boundaries. In order to not just jump across boundaries, Δ*x_max_* has to be smaller than the smallest object (similar to microscopes, where the wavelength determines the spatial resolution). The normal distribution, in contrast, should not be truncated to less than 3*σ*, which means that $\Delta x_{max,n}=3\sqrt{2D\Delta t_{n}}=\sqrt{18D\Delta t_{n}}$. If the step length decision is determined by the spatial aspects as indicated above, then a normal distribution requires Δ*t_n_* ≤ 1*/*3Δ*t_u_* (depending on the truncation). Taking also the higher costs of normally distributed random numbers into account, a simulation with uniformly distributed random numbers could run about 4 times faster along the simulated time.

Depending on the implementation of the reaction scheme(s), further constraints on Δ*t* can occur. For instance the reaction probability from Equation (9) has to be smaller than 1 (or better 0.2), which leads to

(12) $$\Delta t_{r}\leq \text{min}\left(\frac{4\pi {\left(r_{i}+r_{j}\right)}^{2}/3}{\kappa_{ij}}\right)$$

Except for strongly diffusion controlled reactions Δ*t_r_* can be much bigger than the constraints from the random walk steps Δ*t_n_* *>* Δ*t_u_*. In order to save computation time on the expensive pair finding for bimolecular reactions, reactions can thus be executed with a lower frequency, while diffusion steps executed with uniformly distributed random walk steps at higher frequency can converge to the normal distribution and correctly sample the details of the spatial structures in the cell. Likewise this approach allows refilling of reaction volumes between two reaction steps to the local concentration, which is necessary to obtain the correct reaction rate [75].

## 3. Applications and Results of Spatial Simulations

### 3.1. Current Spatial Simulation Frameworks for the Cellular Level

Table 2 lists current spatial simulation packages. The methods employed are mostly particle based (Brownian/Smoluchowski or Green’s function reaction dynamics), or lattice based using ODE/PDE/SDE methods. Due to its relevance, we will describe Gillespie’s stochastic simulation algorithm for the chemical master equation (CME) or the spatial version thereof (reaction diffusion master equation, RDME) in the next section.

Further methods that have been used for cell simulations are for instance: dissipative particle dynamics [151], an Ising model to calculate the ability of gradient sensing in the presence of multiple competitive ligands [152], cellular Potts models [153], or lattice based methods where the lattice sites have the size of molecules [105]. Special multi-scale methods have been developed to accommodate the heterogeneous process of the cells like crowding with small and large molecules [154] or slow and fast reactions [155], which also involves for instance local mean field closures for interactions [156] and coupling of several simulation methods [14].

Parallelization for all these methods has been investigated, in order to increase the performance [107,133,157,158]. Note that the computational particle object does not necessarily have to coincide with an atom, molecule, or other cellular object. It can also represent a subvolume of the cell or the membrane, exchanging mass or other content with neighbours, interact and evolute as described by additional rules [150]. Thus particle or agent based simulations can be used for many simulation tasks in biology [30].

### 3.2. The Reaction Diffusion Master Equation and Gillespie’s Algorithm

The chemical master equation CME or reaction diffusion master equation RDME if space is considered describes the changes in a reaction system consisting of *M* molecular species and *K* reaction channels with rate constants ***k*** = (*k*_1_*, . . ., k_K_*)*^T^* [20]. The reactions take place in reaction compartment Ω. For a spatially resolved description Ω can be subdivided into *U* subvolumes of volume *V*_1_*, . . ., V_U_* [144,157,159–161]. Here we denote the number of particles in subvolume *ν* with ***N****^ν^*(*t*) = (*N*_1_*^ν^* (*t*)*, . . ., N_M_^ν^* (*t*))*^T^*. Time evolution on this level is driven by Markovian population dynamics. More specifically, the probability distribution *P*(***N***^1^(*t*) = ***n***^1^*, . . .,* ***N****^U^*(*t*) = ***n****^U^*) = *p*(***n***^1^*, . . .,* ***n****^U^, t*) satisfies the RDME

$$\frac{\partial }{\partial t}p(n^{1},\ldots ,n^{U},t)=(R+D)p(n^{1},\ldots ,n^{U},t)$$

where *ℛ* and 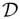 are the reaction and diffusion operators, respectively. The definition of the reaction operator follows from the classical master equation and thus reads

$$R=\sum_{\nu =1}^{U}\sum_{j=1}^{K}\left({\text{E}}_{\nu }^{-\delta_{j}}-1)a_{j}(n^{\nu }\right)$$

where ***δ****_j_* denotes the stoichiometric change vector associated with the *j*-th reaction. This change is performed by the shift operator E*_ν_* that applies to everything to its right-side. It is defined as **E***_ν_*^***δ**_j_*^ *f*(***n***^1^*, . . .,* ***n****^U^, t*) = *f*(***n***^1^*, . . .,* ***n****^ν^* − ***δ****_j_, . . .,* ***n****^U^, t*) for any function *f* of appropriate domain—in particular *f*(***n***^1^*, . . .,* ***n****^U^, t*) = *a_j_*(***n****^ν^*)*p*(***n***^1^*, . . .,* ***n****^U^, t*). Diffusion of species *i* with diffusion coefficient *D_i_* into another volume μ is translated into a first-order transport reactions with effective propensity *ki^ν^*^→^*^μ^n_i_^ν^*. The corresponding rate constant can be expressed as

(13) $$k_{i}^{\nu \to \mu }=\frac{D_{i}}{l^{2}}=\frac{D_{i}\times l^{2}}{l\times l^{3}}=\frac{D_{i}S_{\nu ,\mu }}{lV_{\nu }}$$

where *S_ν,μ_* is the surface/interface area of the cubic subvolumes. Thus, the diffusion operator 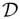 reads

$$D=\sum_{\nu =1}^{U}\sum_{\mu \in N(\nu )}\sum_{i=1}^{M}({\text{E}}_{\nu }^{\Delta_{i}}{\text{E}}_{\mu }^{-\Delta_{i}}-1)k_{i}^{\nu \to \mu }n_{i}^{\nu }$$

where **Δ***_i_* is the vector of change caused by the transport of a single molecule of type *i* from one volume to another one. The set 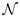
(*ν*) denotes the volumes *μ* in the neighborhood of volume *ν*.

In terms of simulating the reactions the waiting time *τ_j_^ν^* for every reaction *j* within volume *ν* is distributed exponentially with parameter *a_j_*(***n****^ν^*), called the propensity of reaction *j* within *ν*. The waiting time *τ^ν^* for any reaction to occur in *ν* is exponentially distributed according to parameter $a_{0}(n^{\nu })=\sum_{j=1}^{K}a_{j}(n^{\nu })$. Starting from a given time *t*, the next event of reaction *j* in *ν* is according to Gillespie’s algorithm [20] at

(14) $$t_{j}^{\nu }=t+\tau_{j}^{\nu };\;\;\;\tau_{j}^{\nu }\sim \text{Exp}(a_{j})$$

A transport reaction into *μ* at time *t*′ causes a state change ***N****^μ^*(*t*) → ***N****^μ^*(*t*′), which according to Gillespie’s algorithm would require updating the precomputed waiting times *t*_−_*^μ^* in *μ*. Anderson [162] proved that the remaining fraction of the time to the next reaction can simply be stretched according to the changed propensity

(15) $$t_{+}^{\mu }=t^{\prime }+(t_{-}^{\mu }-t^{\prime })\times \frac{a_{0}(N^{\mu }(t))}{a_{0}(N^{\mu }(t^{\prime }))}$$

Of course there exist other ways in implementing the simulation, because the next reaction time Equation (14) can either be calculated cumulatively for all reaction based on *a*_0_ or individually for *a_j_^ν^* for each subvolume and reaction channel. Note that the minimum waiting time of all individual waiting times will be distributed as *Exp*(*a*_0_) such that both descriptions are equivalent, and actually any partitioning/grouping of reaction channels is possible in order to improve the execution performance [155]. Individual reactions have to be executed in their order in time, while the resulting changes in ***N****^ν^*(*t*) can require updates in other waiting times as in Equation (15). If the waiting time is calculated based on the cumulative *a*_0_ instead, the reaction channel and compartment of the next reaction have to be found based on their individual probabilities *a_j_^ν/^a*_0_. Simulators based on Gillespie’s algorithm for the RDME are for example MesoRD [143] or STEPS [146] (cf. Table 2). The discretization into the subvolumes should not be too small such that the chance of two reactants being in the same subvolume goes to zero. In that case simulations of the RDME become diffusion limited [163–165].

Note that for a conversion from particle based simulations to population level of the *i^th^* species in *ν* all particles of that species with ***x***(*t*) ∈ *V^ν^* have to be counted. Conversely, the underlying assumption of the population dynamics model is that the *N_i_^ν^* (*t*) molecules are uniformly distributed in *V^ν^*.

### 3.3. Rule-based Modeling

The different phosphorylation levels as well as the localization of signaling molecules creates a multitude of states in a species/population based description of the system. The number of reactions and rate constants that have to be determined increases even more dramatic, because two interacting proteins each with *n* possible states can have up to *n*^2^ different reaction rate constants. In order to be able to generate and analyze detailed models of signal transduction despite this combinatorial explosion, rule based modeling strategies have been developed [166,167]. The syntax/input scheme of such models minimizes/structures the information that has to be entered by using state dependent rules. Based on these rules the models can also be further abstracted, decomposed or reduced for a comprehensive analysis of the complex system [168,169].

The rule-based concept can be easily extended for spatial models, where the particle interactions are now determined by their internal state and the rules assigned [170,171]. Spatial rule-based models can be for instance used to track how simple molecular units self-assemble into large structures [172].

### 3.4. Applications and Results

The following list shows the importance of the mesoscale level in the cell. These applications cover time and length scales above molecular dynamics simulations, though not necessary on the cellular level. Thus they can be used to analyze important questions in molecular and cell biology, ranging from more detailed descriptions of reaction process to the spatial organization of the cell.

- *Binding kinetics and binding sites:* depending on the description level, protein-protein association can become quite complex [36]. For instance if multiple binding sites and diffusion-controlled reactions are considered. Biomolecules can have several binding sites for the same ligand, for instance receptors forming multimers or antibodies [128]. Kang *et al*. [173] analysed this and Park *et al*. [174] developed a theory for reversible reactions under these circumstances. For instance, two binding sites on a molecule would mean that the microscopic reaction rate constant *κ_ij_* is doubled, while the reaction radius is the same as for a molecule with just one binding site. Equation (6) shows that the macroscopic rate constant will not necessarily double under these circumstances.
- *Scaffolds and Channeling:* Both in signaling and metabolic pathways co-localization of related molecules has been observed. Obviously co-localization has advantages because the local high concentration boosts the reaction rate [4,80,106,175,176]. Specific and even nonspecific binding interactions which modify the localization properties of molecules can thus enhance reactions [177]. Note, that the localization requires that molecules do not diffuse around/away, such that there is a trade-off between advantages due to co-localization and disadvantages due to the reduced mobility [75,80].
- *Protein DNA interactions:* Transcription factors have to find their target site on the DNA amongst millions of binding sites, and they do it surprisingly efficiently, e.g., by combining 1D sliding and 3D diffusion [178]. For instance nonspecific interactions could enhance association rates respectively [177]. Note that even DNA is well organized in space [6]. The spatial organization of DNA strands plays an important role, but long DNA strands can obviously not be modeled with full atomic detail in a MDS simulation such that multi-scale approaches have to be employed [25]. The observed bursting kinetics of transcription rates is likewise explained using open and closed chromatin states, which involve large-scale transitions of the DNA state [179].
- *Assembly and fusion processes:* Large polymer structures such as the cytoskeleton filaments play an important role for the spatial organization of the cell. Guo *et al*. modeled the actin assembly using Brownian dynamics [180]. Langevin dynamics have been used to simulate the assembly of virus polymers [181]. A rule-based description was likewise used to analyze the emergence of complex structures [172]. Likewise the fusion of membrane enclosed structures like vesicles is important for the functionality of the cell [64,151,182]. Note that the interplay of cytoskeleton filaments, motor proteins and vesicles can enhance their fusion process [150], while the cytoskeleton structure is for instance organized by the aforementioned growth processes but also motors pulling them together and creating spatial patterns [68].
- *Non-uniform molecule distributions in space:* In order to grow/move in specific directions cells have to polarize into front and back, which is associated with nonsymmetric particle distributions across the cell [134,183]. In addition receptors on the membrane can cluster together [9,184], and the output of spatial simulations shows the importance of the spatial organization in the cell [9]. Note that again reversible binding and/or unspecific binding interactions influence these reaction rates [177,184].

## 4. Towards Multi-Scale Simulations from Atoms to Cells

Simulations on the molecular and cellular level are available and have been used extensively to better understand biological processes [15–19,39]. At least theoretically the different levels can be included in a multi-scale simulation, where all levels are executed in parallel. Starting from a coarse grained Brownian dynamics simulation, more detailed levels could be invoked as soon as two particles are closer than a threshold, e.g., switching from spheres to more realistic shapes [60,185] and eventually resolving each collision on the MDS level.

A sequential execution would probably be easier to implement and thus more relevant in order to model a signaling pathway “ab initio”, starting from the protein sequences of all involved molecules. The corresponding molecular structures can be predicted (at least for small signaling molecules) if not yet known from structure analysis by molecular simulation/analysis tools based on the atomic level [22,186–189].

If possible from these structures the hydrodynamic radii [60,190], binding rates and reaction kinetics can be calculated/estimated [82,188,189,191–198]. Especially important with respect to signal transduction are the conformational changes and their rates associated with “active” and “inactive” states, mostly regulated by the phosphorylation level of the proteins [199]. Based on the diffusion model and the crowding level of the cell, the effective diffusion coefficient can then be estimated (e.g., if crowding should only be modeled implicitly) [12]. The number/concentration of signaling molecules can nowadays be quantified [200] and likewise serves as an input for the simulation [12].

Thus all necessary parameters are available to simulate the spatial and temporal dynamics of a signaling pathway in the cell. The relation of transport and reaction rates beforehand gives an estimator how well mixed the system will be and if the spatial dynamics have to be resolved explicitly [7]. If not, even a population based ODE/SDE model with the effective reaction rates might be sufficient for simulation (of the respective well-mixed subset of the system). But of course the compartmentalization and thus transport rates through nuclear pores/membranes should be included [201].

Given the challenges involved with parameter estimation and model discrimination from experimental results [179,202–205], such a bottom-up simulation approach might bring invaluable insights for the signaling pathway under consideration. Simulations tracking all particle positions inside the cell allow analysis of particle distributions, e.g., to find receptor clusters in the membrane [9,184,206]. Based on such simulations the cell can also be visualized interactively at the desired resolution [11,74,107,129,207], for instance showing local variations in molecule concentrations [207] or even showing the molecules with their atomic structure as in Figure 1. The described bottom-up simulation approach as such cannot include all relevant effects on all levels. Leaving out solvent molecules is the most obvious fact, but intermediary effects like crowding and hydrodynamic forces [12,37] seem to be important. Thus especially efficient yet correct multi-scale simulation algorithms for the mesoscale resolution have to be further developed, and a consensus on the necessary levels of details has to be found.

## Figures and Tables

**Table 1:** Empiric approximations for the hydrodynamic radius *r_h_* based on the molecular weight *MW* in kDa. (i) is a fit to experimental data, e.g., from [69,70]. The other equations assume that the mass is (re-)distributed in a sphere, for instance with a specific volume of 1 cm^3^/g in (ii) [41,71]. Due to the in general nonspherical shape and the “holes” of the molecule, an exponent larger than 1*/*3 as in (i) is reasonable. The hydrodynamic radii reported by [60] fall between (i) and (ii).

	Hydrodynamic Radius [nm]	Reference
(i)	*r_h_* = 0.6169 *× MW*^0.4226^	[72]
(ii)	*r_h_* = 0.7468 *× MW*^1^*^/^*^3^	[41]
(iii)	*r_h_* = 0.5429 *× MW*^1^*^/^*^3^	[40]

**Table 2:** Spatial simulations on the cellular level.

Name/Authors	Features	Website/References
Smoldyn S. Andrews *et al*.	Particle based simulator for reaction diffusion processes in arbitrarily shaped compartments. (point particles, no crowding).	www.smoldyn.org [28,48,119,120,133,134]

ChemCell	Particle based simulator within realistic cell shapes.	chemcell.sandia.gov [93,135,136]

E-Cell	Complete software environment for simulations on several levels. Contains further analysis tools.	www.e-cell.org [137–139]
(GFRD,eGFRD) ten Wolde *et al*.	Green’s function reaction dynamics will be included in the E-Cell project	[110,111,140,141]

FLAME	Agent-based multi-scale simulation (also beyond the cellular level).	www.flame.ac.uk [32,116,117]

MCell	Monte Carlo simulator of reaction diffusion processes. Reactions can only happen at membranes	www.mcell.cnl.salk.edu [142]

MesoRD	Spatial derivative of Gillespie’s algorithm to solve the Reaction-Diffusion Master Equation (RDME) with the “next subvolume method”	mesord.sourceforge.net [143,144]

SmartCell Serrano *et al*.	Spatial derivative of Gillespie’s algorithm in arbitrarily shaped compartments.	software.crg.es/smartcell [145]

STEPS	Tetrahedral mesh based spatial derivative of Gillespie’s algorithm	steps.sourceforge.net/STEPS [146]

STSE S.Stoma	PDE based simulator with compartments and direct linking to microscope images.	www.stse-software.org [147]

V Cell	ODE/PDE or SDE based simulator within realistic cell shapes.	www.nrcam.uchc.edu [148,149]

M. Klann *et al*.	Agent-based Brownian dynamics including cytoskeleton, crowding and vesicle transport.	www.bison.ethz.ch/research/spatial simulations [75,80,106,107,122,150]
